# Unusual Subacute Interphalangeal Tophaceous Gouty Arthritis

**DOI:** 10.7759/cureus.13732

**Published:** 2021-03-06

**Authors:** Ndausung E Udongwo, Mihir Odak, Asseel AlBayati, Min Zheng, Xiaoyin Tang

**Affiliations:** 1 Internal Medicine, Jersey Shore University Medical Center, Neptune, USA; 2 Pathology and Laboratory Medicine, Jersey Shore University Medical Center, Neptune, USA; 3 Rheumatology, Jersey Shore University Medical Center, Neptune, USA

**Keywords:** gout, tophi, hyperuricemia, arthritis

## Abstract

Gout is an arthritic syndrome that causes extensive joint damage and discomfort. It is due to an elevated uric acid level in the blood which deposits in the joints. This causes an inflammatory response and joint damage. Gout typically presents as an acute monoarticular attack, resulting in hot, erythematous, swollen, and exquisitely tender joint. Tophaceous gout, which is commonly a later complication of long-standing gout, can rarely be the presenting manifestation of gout.

Tophaceous gout is considered a late complication of chronic gout. As early recognition of gout can lead to prompt initiation of treatment and improvement in clinical status, a patient with an alcohol use history who presents with polyarticular swelling and tenderness should raise the suspicion of a tophaceous gout exacerbation even if the patient does not have a documented history of gout.

## Introduction

Gout is a debilitating inflammatory syndrome that can afflict patients with severe pain and joint dysfunction. Over the last 30 years, the prevalence of gout in the United States has increased from 2.9 to 37.6 cases out of every 1,000 people, representing a drastic increase in the number of gout cases [1]. Gout happens to be more prevalent in men as compared to women, and evidence has found a predilection for individuals of advancing age to be affected more than youth; despite this, the rising incidence and prevalence of gout indicates a deficiency in management abilities [2]. Gout is caused by a build-up of uric acid in the blood, termed hyperuricemia, and subsequently in the joints, leading to inflammation and severe pain, joint dysfunction, and deformity [3]. This hyperuricemia can be due to overproduction of uric acid, in conditions that cause widespread inflammation and tissue damage or secondary to a diet that predisposes to hyperuricemia, or decreased excretion of uric acid from the body in the form of renal dysfunction. The typical presentation of gout involves an acute onset of erythema, swelling, and tenderness of the affected joint. The most commonly affected joint is the first metatarsophalangeal joint [3,4]. A complication of gout is the deposition of uric acid in tissues, known as tophaceous gout [4].

Here we present a case of a 64-year-old female with no prior history of gout who complained of painful joint swelling of the distal interphalangeal joint. She was initially being treated for a possible septic joint. However, due to unresponsiveness, surgical evaluation of the hand raised suspicion for tophaceous gout. We hope this clinical scenario highlights the non-classical presentations of gout, as early diagnosis and therapy can be significantly different and may induce timely remission and prevent unnecessary interventions.

## Case presentation

**Figure 1:**
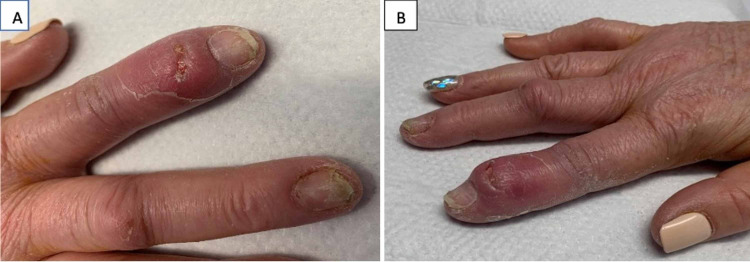
Right index finger: distal interphalangeal joint inflammation (A and B).

**Figure 2:**
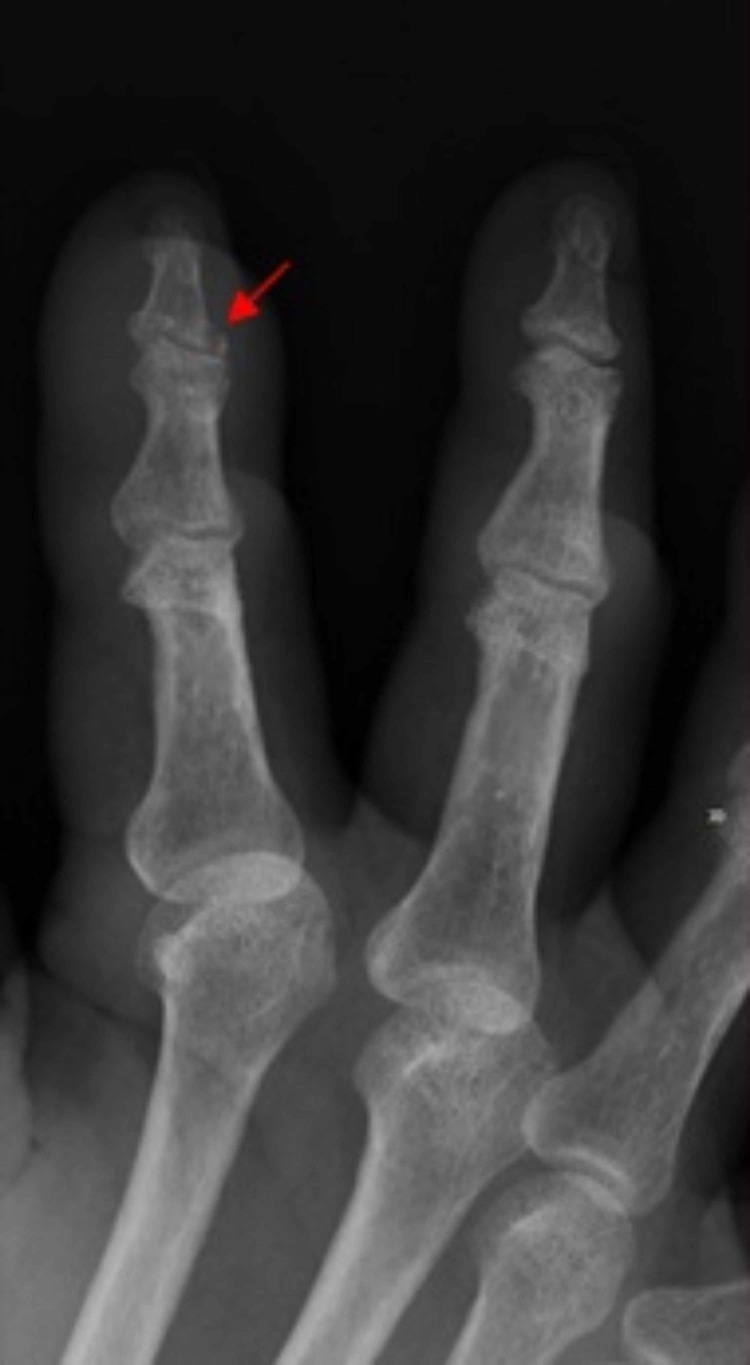
Anteroposterior radiographic imaging of the right hand, showing the distal interphalangeal joint with a classical bony erosion (red arrow).

**Figure 3:**
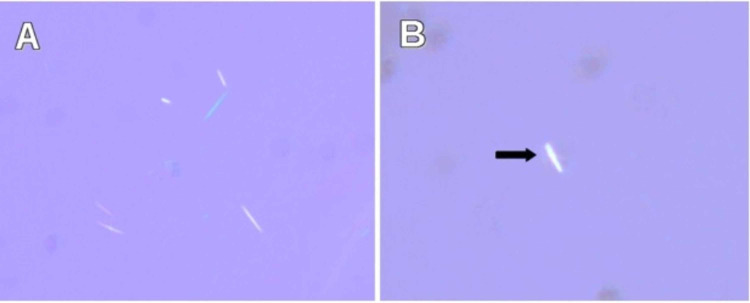
Identification of uric acid crystals with polarized microscopy. (A) Aspirated joint fluid shows scattered needle-shaped, negative birefringent monosodium urate crystals (x400). (B) Some crystals appear intracellular (arrow; x630).

We present a case of a 64-year-old female who was readmitted to the hospital with complaints of worsening pain in her fingers. Her symptom was associated with fever, as well as swelling around the right index and middle fingers. She was recently discharged to a rehabilitation facility after being treated for humeral head and femoral neck fracture which was sustained from a fall. During her prior hospitalization, she endorsed drinking two to three glasses of wine daily, which was confirmed with a blood alcohol level of 0.263%. She had a past medical history of breast cancer in remission, osteoporosis, hypertension, hyperlipidemia, hypothyroidism, and chronic anemia (iron and vitamin B-12 deficient). She was a former tobacco user. Her family history was non-contributory. Her oral medications were metoprolol succinate 25 mg daily, oxycodone 5 mg every four hours as needed, iron polysaccharide 150 mg daily, cyanocobalamin 100 mcg daily, levothyroxine 175 mcg daily (before breakfast), and atorvastatin 10 mg daily. She endorsed having a previous episode in the past (one year ago) and was treated for suspected methicillin-resistant *Staphylococcus aureus* (MRSA) induced septic arthritis. On physical examination, her vitals were as follows: blood pressure of 110/68 mmHg, a temperature of 100. 5°Fahrenheit, a heart rate of 104 beats per minute, a respiratory rate of 18 breaths per minute, and oxygen saturation of 96% on room air. There was edema, redness, and purulent-like drainage on the right index finger (Figure 1). Pain on movement and palpation of the right index and middle fingers was appreciated, as compared to the left. The right upper extremity was immobilized with a sling. There were no murmurs, rubs, or gallops on auscultation of the heart. Also, there was no wheeze, rales, or rhonchi on auscultation of the lungs. Laboratory results showed hemoglobin of 8.1 g/dL (normal range: 12-16 g/dL), white blood cell count of 10.9 x 10^3^/uL (normal range: 4.5-11 x 10^3^/uL), sodium of 135 mmol/L (normal range: 136-145 mmol/L), potassium of 3.6 mmol/L (normal range: 3.5-5 mmol/L), blood urea nitrogen of 8 mg/dL (normal range: 5-25 mg/dL), and creatinine of 0.97 mg/dL (normal range: 0.44-1.0 mg/dL). Blood and wound cultures were sent to the laboratory. She was again admitted for suspected MRSA-induced septic arthritis and started on intravenous vancomycin with pain management. X-ray of the right hand revealed soft tissue swelling of the second and third fingers, with a small erosion at the base of the distal phalanx (index finger) (Figure 2), raising concerns for osteomyelitis. Limited results from magnetic resonance imaging (MRI) of the right hand, with and without contrast, showed possible osteomyelitis only involving the proximal and distal interphalangeal joints (PIP/DIP) of the index finger. Due to the worsening of her symptoms, she was eventually taken to a surgical room by the hand surgeon for tissue biopsy, as well as incision and drainage. The area of greatest fluctuance was mapped out and then an incision was made with a 15-blade scalpel. Purulent-like drainage with tophaceous-type calcification was appreciated. Eventually, blood cultures and wound cultures came back negative (no growth after five and two days, respectively). The tissue biopsy showed synovial fibrous tissue containing amorphous material surrounded by granulomatous inflammation, and the synovial fluid direct smear analysis showed characteristic needle-shaped negative birefringent uric acid crystals under polarized light microscopy (Figure 3). Uric acid level was 8.6 g/dL (normal range: 4-8 g/dL). In addition, she was found to have pain and edema on the right knee and the left ankle, as revealed by a detailed examination performed by a rheumatologist. Non-steroidal anti-inflammatory drugs (NSAIDs) and steroids were deferred due to worsening kidney function and recent history of fractures. She was started on oral colchicine 0.6 mg daily, and her symptoms improved.

## Discussion

Crystal-induced arthropathies (CIAs) are groups of inflammatory disorders affecting articular or periarticular surfaces in the body. Symptoms could range from mild pain to excruciating joint pain due to the destruction of articular surfaces, causing both physical and psychological problems in affected individuals. In the United States, gout is still regarded as the most common type of CIA (3-4% in prevalence) as compared to basic calcium phosphate, calcium pyrophosphate dihydrate deposition, and calcium oxalate crystal arthropathies [1,5]. Predominantly, it affects men more than women (prevalence of 9% and 8%, respectively) [6], with the former frequently seen in ages greater than 50 years (post-menopause) [6]. The proposed mechanism could be secondary to increased uric acid excretion caused by the estrogen effect in the renal tubules of premenopausal women [6,7].

Risk factors include modifiable (lifestyle and diet) and non-modifiable (age, sex, and race) factors. Also, comorbid diseases such as diabetes, hypertension, and hyperlipidemia, as well as certain medications are also crucial in increasing the risk of this disease. Gout is the result of a derangement in uric acid metabolism either from overproduction, decreased excretion (kidney disease), or increased consumption of purine-rich foods [8,9]. This could lead to uric acid accumulation in articular or periarticular surfaces, which would trigger an inflammatory response leading to acute, intercritical, or chronic gout symptoms. “Podagra'' is a common presenting symptom in acute gout attacks, where there is an inflammation of the metatarsophalangeal joint of the first big toe. Monoarticular involvement is a common presentation in acute gout attacks [10]. Tophaceous gout, on the other hand, is regarded as a late complication of chronic gout [10]. It rarely presents as an acute gout attack and is an uncommon presentation in the hands. However, in an article published by Fitzgerald et al., it can be seen as the first sign of gout, most especially in elderly women and appearing more on fingers and wrists [6]. Due to its rare acute presentation, most cases are misdiagnosed or thought to be ongoing septic arthritis or cellulitis. Thus, causing either a delay in management or inappropriate exposure to antibiotics, thereby increasing resistance to and other side effects of medications [11].

A proper diagnosis of gout requires a good history and physical examination, laboratory results, and imaging studies. Fluid aspiration from affected joints is still known to be the gold standard test in confirming this disease, with a sensitivity of 84.4% and a specificity of 97.2% [9]. In cases where there is a presence of suspected tophi deposits on finger pads, fluid aspiration and crystal analysis are recommended [11].

The formation of urate crystals is still not clearly understood, but it has been linked to certain factors such as temperature and pH changes. Once they are formed, phagocytic macrophages engulf this foreign body, triggering an immune response releasing cytokines (tumor necrosis factor-alpha, interleukin-6, and complement factors) and activating neutrophils. With the activation of neutrophils, the inflammatory response around deposited urate crystals (articular or periarticular surfaces) will lead to the type of symptoms experienced by our patient [10-13].

Fitzgerald et al. described tophaceous materials presenting as three different morphologies: (a) acutely, presenting in liquid form, (b) subacutely, presenting in a thick or more viscous form, and (c) chronically, presenting as chalky or granular form. The subacute form is similar to the purulent discharge seen in our patient [6]. A contributing factor to tophaceous material deposition on fingers is the reduced or cooler temperature in this area.

Shmerling et al. described four cases (between 76 and 86 years of age) with tophi deposition in finger pads and no prior history of gout. They all presented with arthritic symptoms, suspicious of either cellulitis or osteoarthritis. They were all postmenopausal, with a history of hypertension treated with diuretics, no reported family history of gout, and increase uric acid levels. Two out of the four received inappropriate management initially until gout was confirmed. The 76-year-old female who had a history of hypertension, which was controlled with hydrochlorothiazide and propranolol, had a close similarity to our patient. She had two previous episodes in the past, but it was misdiagnosed as cellulitis. Just like our patient, she received intravenous antibiotics. Eventually, she received the appropriate treatment after gout was diagnosed, and her symptoms resolved. In comparison to our case, our patient reported consuming wine daily (two to three glasses per day) but had no diuretic use. Also, she reported having only one previous episode and was treated for suspected MRSA infection of the joint. It took more than three days to eventually diagnose tophaceous gout in our patient. Unfortunately, she developed a worsening of her kidney function, partly due to the intravenous vancomycin she received empirically. Colchicine was the only acceptable pain medication due to her borderline renal function and recent bone fractures, which were contraindications to either NSAIDs or steroid [11].

To our knowledge, there are only a few reported cases of this disease presentation in literature. We hope more cases be reported to help in early recognition, initiation of the appropriate treatment, and preventing complications of other unnecessary interventions.

## Conclusions

An initial presentation of a subacute attack of gout in the interphalangeal joints is rarely seen. Due to the rarity of this disease, there is delay in the diagnosis and management. Tophaceous deposits have been misdiagnosed as cellulitis or septic joint, just like in our case. Our aim is to create more awareness, which we believe will help limit delay in diagnosis and management of this obscured gout presentation. There should be high suspicion for gout when patients, usually in this age group, especially with risk factors such as alcohol use and hypertension, present with arthritic symptoms and unusual finger deposits. This highlights the need for more cases to be reported in the literature, which would help improve quality of life of these patients.
